# MPC-STANet: Alzheimer’s Disease Recognition Method Based on Multiple Phantom Convolution and Spatial Transformation Attention Mechanism

**DOI:** 10.3389/fnagi.2022.918462

**Published:** 2022-06-10

**Authors:** Yujian Liu, Kun Tang, Weiwei Cai, Aibin Chen, Guoxiong Zhou, Liujun Li, Runmin Liu

**Affiliations:** ^1^College of Computer and Information Engineering, Central South University of Forestry and Technology, Changsha, China; ^2^School of Artificial Intelligence and Computer Science, Jiangnan University, Wuxi, China; ^3^AiTech Artificial Intelligence Research Institute, Changsha, China; ^4^Department of Civil, Architectural and Environmental Engineering, Missouri University of Science and Technology, Rolla, MO, United States; ^5^College of Sports Engineering and Information Technology, Wuhan Sports University, Wuhan, China

**Keywords:** MPC-STANet, Multi-Phantom Convolution, Space Conversion Attention Mechanism, Synthetic Minority Over-sampling Technique, Alzheimer’s disease recognition

## Abstract

Alzheimer’s disease (AD) is a progressive neurodegenerative disease with insidious and irreversible onset. The recognition of the disease stage of AD and the administration of effective interventional treatment are important to slow down and control the progression of the disease. However, due to the unbalanced distribution of the acquired data volume, the problem that the features change inconspicuously in different disease stages of AD, and the scattered and narrow areas of the feature areas (hippocampal region, medial temporal lobe, etc.), the effective recognition of AD remains a critical unmet need. Therefore, we first employ class-balancing operation using data expansion and Synthetic Minority Oversampling Technique (SMOTE) to avoid the AD MRI dataset being affected by classification imbalance in the training. Subsequently, a recognition network based on Multi-Phantom Convolution (MPC) and Space Conversion Attention Mechanism (MPC-STANet) with ResNet50 as the backbone network is proposed for the recognition of the disease stages of AD. In this study, we propose a Multi-Phantom Convolution in the way of convolution according to the channel direction and integrate it with the average pooling layer into two basic blocks of ResNet50: Conv Block and Identity Block to propose the Multi-Phantom Residual Block (MPRB) including Multi-Conv Block and Multi-Identity Block to better recognize the scattered and tiny disease features of Alzheimer’s disease. Meanwhile, the weight coefficients are extracted from both vertical and horizontal directions using the Space Conversion Attention Mechanism (SCAM) to better recognize subtle structural changes in the AD MRI images. The experimental results show that our proposed method achieves an average recognition accuracy of 96.25%, F1 score of 95%, and mAP of 93%, and the number of parameters is only 1.69 M more than ResNet50.

## Introduction

Alzheimer’s disease (AD) is an insidious and slowly progressive neurodegenerative disease, which is mainly found in the elderly population over 60 years of age and is clinically manifested as amnesia, loss of mobility, language ability, etc. (Beitz, 2014; Andrieu et al., 2015). Alzheimer’s disease has a long developmental cycle and is divided into five disease stages: Non-Demented, Very Mild Demented, Mild Demented, Moderate Demented, Severe Dementia. Very Mild Demented, where people with Mild Demented often have memory loss, and in severe cases, dementia; Mild Dementia, where people show a lack of memory, personality changes, disorientation, and difficulty performing daily tasks; Moderate Dementia, where patients experience significant personality changes and sleep disturbances, and already require additional care and support, which can be easily recognized by health care professionals. Severe Dementia, where patients with this condition already lack the ability to communicate, have difficulty completing the small tasks of life and require full-time treatment. Due to the long stage of Alzheimer’s disease and the lack of obvious changes in the features of the early disease, it is difficult for patients themselves to realize this and it is difficult for doctors to make a correct judgment in time based on some of the small pathological features of patients in the early stages of the disease (the first four disease stages of Alzheimer’s disease) (Wu and Swaab, 2005). When the symptoms of patients are obvious before they are diagnosed, Alzheimer’s disease has already reached the late stage (the fifth disease stage: Severe Dementia). At this time, the patient has the problems of being unable to eat and incontinence and needs others to take care of their daily life, including eating or going to the toilet. A large number of nerves in patients have experienced irreversible death, and the reflex becomes abnormal, resulting in irreversible cognitive degeneration and dementia, which cannot achieve good therapeutic effects (Nelson et al., 2012). The use of deep learning research has little significance in recognizing severe dementia. Therefore, in this study, we only carry out diagnosis and recognition for the first four stages of Alzheimer’s disease, which is of great significance for slowing and controlling the progress of the disease (Dubois et al., 2016).

The pathogenesis of Alzheimer’s disease is complex, among which age is an important factor in the cause of this disease, and genetic factors, external trauma, education level, trace elements, etc., are also important reasons for the occurrence of this disease (De la Torre, 1999). The biological features of Alzheimer’s disease include the formation of senile plaques due to the accumulation of β-amyloid (Aβ) in the cerebral cortex and the hippocampal region, neuronal cell reduction, and neurofibrillary tangles within neuronal cells, etc. (Zhao and Zhao, 2013). The brain structure of Alzheimer’s disease patients is mainly characterized by brain atrophy, narrowing of the gyrus, enlargement of the sulcal gaps, and the degree of atrophy in the hippocampus region and medial temporal lobe atrophy compared to normal people. The observation of the brain structure of Alzheimer’s patients is mainly through the Alzheimer’s MRI medical images, which capture information about the relevant disease pattern of the patients through neuroimaging of the white matter area of the brain and assist doctors in judging the disease stage of Alzheimer’s disease, while the Alzheimer’s MRI medical images have the problems of difficulty in acquiring and the extremely unbalanced distribution of the acquired data volume (Chen and Glover, 2015). The manual recognition process of Alzheimer’s disease is very complex. First, doctors need to ask the patient about recent living environment through psychological scales to assess whether his/her cognitive functions have deteriorated, then employ nuclear magnetic imaging to check whether the imaging structures of the brain of the patient have started to atrophy and change, and finally use electroencephalogram and long-term monitoring of the heartbeat to determine whether the patient is showing changes in cognitive functions and brain signals. Such a testing process relies on the professional knowledge of the physicians and clinical experience, but manual analysis of the medical image is time-consuming and laborious, and there is a risk of misdiagnosis. Therefore, if we can employ a computer to assist in diagnosis, we can improve the efficiency of doctors to a certain extent and also reduce the misdiagnosis and leakage caused by humans (Frisoni et al., 2010; Royce et al., 2019; Lu et al., 2020).

The stage recognition of Alzheimer’s disease has been a popular research direction in the field of computer vision-aided diagnosis, and numerous studies have combined traditional machine learning methods to recognize this disease and achieved good recognition results (Negin et al., 2018; Wang et al., 2019). For example, Magnin et al. (2009). proposed and evaluated a novel automated method of whole-brain anatomical MRI based on support vector machine (SVM) classification to distinguish Alzheimer’s disease (AD) patients from elderly control subjects, with a mean correct classification of 94.5% (mean specificity 96.6%; mean sensitivity 91.5%) for AD and control subjects. Lebedev et al. (2014) used a random forest classifier trained based on MRI measures of different structures for the diagnosis of Alzheimer’s disease and achieve the best AD/HC sensitivity/specificity (88.6%/92.0%) results after combining with cortical thickness and volume measurements. However, although the above research methods were successfully applied to Alzheimer’s disease classification and diagnosis, the extraction of effective features in Alzheimer’s disease diagnosis often plays a more important role than the construction of classifiers, which requires manual selection of regions of interest before classification and a series of manual feature extraction steps with *a priori* knowledge, which is a tedious extraction process and has human factors interfering (Li et al., 2012; Sabuncu and Konukoglu, 2015). With the development of computer platforms, convolutional neural networks (CNNs) have been widely recognized for their good image recognition, and a large number of CNN-based Alzheimer’s classification models have emerged. For example, Sarraf and Tofighi (2016) used convolutional neural networks to successfully classify functional MRI data from Alzheimer’s brains with normal healthy brains, where the accuracy of the test data reach 96.85%. Ieracitano et al., 2019 proposed a data-driven approach to distinguish subjects with AD, MCI, and HC by acquiring electroencephalogram recordings and transforming the correlation spectra of 19 channels of electroencephalogram traces into 2D grayscale images, and then classifying binary and multiple classes in 2D images using CNN models with 89.8 and 83.3% accuracy, respectively.

The above examples all show the application results of the field of deep learning in Alzheimer’s disease well while demonstrating the better adaptability and data discrimination of the convolutional neural networks (CNN) within the field of Alzheimer’s disease recognition. However, due to the complex structure of the human brain during Alzheimer’s disease and the difficulty of detecting subtle structural changes in the brain during mild disease, and the fact that the aging process of normal people is accompanied by shrinkage of brain structures, Alzheimer’s patients also suffer from shrinkage of brain areas, which poses many difficulties for research (Young et al., 2013; Lockhart and DeCarli, 2014). Only the correct determination of the changes in brain structure can effectively diagnose the different stages of Alzheimer’s disease. Therefore, the main problems of this study are as follows: (1) the Alzheimer’s MRI medical images acquired during the first four disease stages of Alzheimer’s disease have the problem of unbalanced distribution in terms of data volume, which can affect the training effect of the model and make the classification results biased toward the class with more MRI images. (2) The brain structures in different disease stages of Alzheimer’s disease produce subtle changes on MRI images, and the regions of interest (e.g., sulcal gaps, gyrus, hippocampal region, medial temporal lobe) account for a small proportion of the whole MRI image, complicating feature extraction. (3) The lack of distinctive features of Alzheimer’s disease makes convolutional neural networks often accompanied by an increase in the number of convolutional layers to improve the ability of the neural network for feature extraction. However, when the number of layers of the neural network exceeds a certain threshold, there will be problems such as gradient disappearance and gradient explosion, making the neural network difficult to be trained, and the long-time training is not conducive to Alzheimer’s disease prediction (Guo et al., 2017; Wu et al., 2018; Hu et al., 2021).

To deal with the problem of classification imbalance in the dataset, the most basic approach is either to directly copy the minority classes and add them to the sample set or to employ a certain percentage of the majority classes as the training set to obtain a relatively balanced dataset (López et al., 2013; Maxwell et al., 2018). However, this approach tends to lead to the problem of model overfitting, which makes the information learned by the model not generalized enough. To address this problem, Chawla et al. (2002) proposed the Synthetic Minority Oversampling Technique (SMOTE), which uses the similarity between the classes with fewer samples in the feature space to build synthetic new samples and add them to the minority classes. The SMOTE is a good solution to the problem that the information obtained by random oversampling is too special and not generalized enough. Therefore, we combine SMOTE with data expansion (flipping, adding random Gaussian noise, and contrast adjustment) for Alzheimer’s disease to perform class-balancing preprocessing for better training results.

Given the little variation in the Alzheimer’s MRI images in different disease stages and the small proportion of regions of interest, Toğaçar et al. (2021) used DeepDream, fuzzy color image enhancement, and super columnar techniques to process the Alzheimer’s MRI dataset, input the processed MRI dataset into VGG-16 for feature extraction, and finally used Support Vector Machine (SVM) as a classifier. The recognition accuracy of using this method was 100% for MD and ND as well as 99.94% for VMD and MOD. Using the data enhancement algorithm on the Alzheimer’s MRI dataset can enhance the features of each MRI image and suppress useless background information so that the deep learning model can better extract these features and achieve a high recognition rate. However, the use of data enhancement algorithms often has the problems of a cumbersome operation process and poor generalization ability, and the input of the deep learning model often requires a large amount of image data, which takes a long time to complete the feature enhancement operation. Thus, we improve the ability of the network to extract features by redesigning the structure of the deep neural network to avoid using more feature enhancement algorithms for the dataset. Therefore, we propose a recognition network of Alzheimer’s disease based on Multi-Phantom Convolution and Space Conversion Attention Mechanism (MPC-STANet) with the residual network ResNet50 as the backbone network (He et al., 2016). Compared with VGG-16, ResNet50 has lower complexity and required parameters, and faster convergence speed. It has 50 training layers, which can extract more subtle features from Alzheimer’s MRI images, with better classification accuracy. Moreover, the unique residual connection of ResNet50 breaks the symmetry of the neural network, improves the utilization of neurons in each layer, and multiple branches ensure that even if some layers degenerate, it will not affect the overall performance, which makes it widely used in the field of image recognition. Since the feature performance of the Alzheimer’s MRI medical image is very different from that of an ordinary image, we structurally designed the convolutional layer of ResNet50 to better suit Alzheimer’s disease: (1) To increase the feature extraction of small and scattered regions in the Alzheimer’s MRI images, we employ dilated convolution (Yu and Koltun, 2015) instead of the original 7 × 7 convolution layer in STAGE1 to obtain a larger perceptual field without changing the number of parameters. (2) To extract more subtle pathological features of structures, we employ Multi-Phantom Convolution and the Space Conversion Attention Mechanism in the residual blocks to extract richer characterization information from patients’ MRI images. Meanwhile, to avoid the redundancy of useless information, we add an average pooling layer to integrate space information in the shortcut branch of the residual block to improve the detection speed along with reducing the computation.

The contributions of this study are as follows.

**FIGURE 1:**
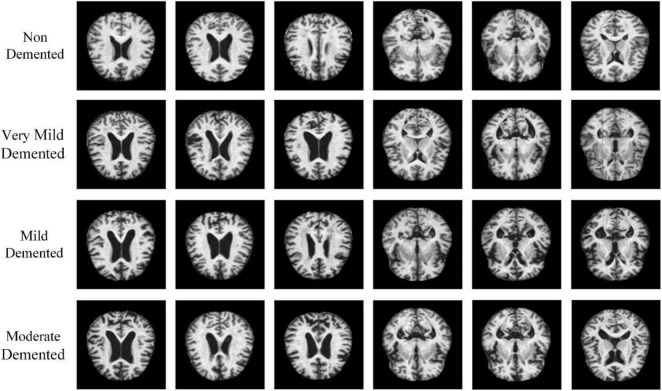
Principles of Alzheimer’s disease recognition.

**FIGURE 2:**
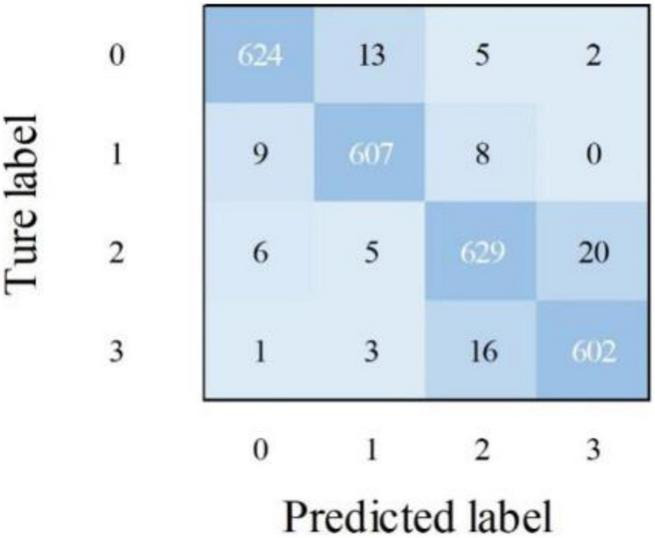
Confusion matrix of the MPC-STANet.

(1) To solve the problem of classification imbalance in the Alzheimer’s disease dataset, we increased the data volume in the minority classes using data expansion methods such as flipping, adding noise, and contrast adjustment (as depicted in Figure 1), and performed class-balancing operation using SMOTE (the results are displayed in Table 1). SMOTE performs a class-balancing operation by artificially synthesizing new samples from the minority classes and adding them to the dataset for classification balance, which well solves the problem of model overfitting, as displayed in Table 2. In the MPC-STANet model, the recognition accuracy of the class-balancing processed dataset is improved by 10.7% compared to the unprocessed dataset.

(2) To extract the scattered and subtle pathological features of Alzheimer’s disease, the MPC-STANet is proposed in this study. (a) We employ Dilated Convolution in STAGE 1 of the network to extract features from scattered pathological regions of Alzheimer’s disease to obtain a larger range of feature information. As displayed in Table 3, the recognition accuracy of ResNet50 after using Dilated Convolution is improved by 2.1% compared with ResNet50. (b) To extract more subtle pathological features, we proposed Multi-Phantom Residual Block (including Multi-Conv Block and Multi-Identity Block) based on Multi-Phantom Convolution, average pooling layer, and Conv Block and Identity Block of ResNet50 to extract richer characterization information in the Alzheimer’s MRI images, and the recognition accuracy is improved by 4.9% compared to ResNet50 and the model parameters decreased by 4.46M compared to ResNet50 (as displayed in Table 4). (c) Space Conversion Attention Mechanism is inserted between Multi-Phantom Convolution and 1 × 1 convolution, aiming to solve the problem of difficult recognition due to small differences between disease stages. Space Conversion Attention Mechanism preserves more important feature information (e.g., hippocampal region, brain gyrus, sulcal gaps, etc.) and discards redundant information (e.g., background) by assigning different weights in vertical and horizontal directions to enhance the extraction of tiny features, and the recognition accuracy is improved by 5.5% compared to ResNet50 (as displayed in Table 5).
(3) The recognition accuracy of the recognition methods proposed in this study for the first four stages of Alzheimer’s disease in non-demented, very mild demented, Mild Demented and Moderate Demented are 97, 95, 98, and 94%, respectively. Other performance evaluations are shown in Table 6 and the confusion matrix of the MPC-STANet is shown in Figure 2. In experiment 3.5, we tested the MPC-STANet and other networks in the same environment. The experimental results show that the Recall, F1-score, Precision, and mAP of the MPC-STANet proposed are 96, 95, 96, and 93%, respectively, which are higher than the other networks. The overall performance of the model is good, and the performance evaluations of other networks are shown in Table 7.

**TABLE 1:** The number of the four disease stages and their proportions.

Disease type	Original number	Percentage	Expanded number	Percentage
Non-Demented	3200	50%	3200	25%
Very Mild Demented	2240	35%	3200	25%
Mild Demented	896	14%	3200	25%
Moderate Demented	64	1%	3200	25%

**TABLE 2:** The recognition accuracy of the original dataset and the preprocessed dataset in the three models.

Network model	Original data set	Preprocessed data set
ResNet50	76.9%	84.6%
ResNet50-SPAM	81.2%	89.4%
MPC-STANet	85.5%	96.2%

**TABLE 3:** Comparison of accuracy and number of parameters of four networks.

Network model	Parameters	Accuracy
ResNet50	25.56M	84.6%
ResNet50-DC	25.56M	86.7%
MPC-STANet	27.25M	96.2%

**TABLE 4:** Comparison of accuracy and number of parameters of three networks.

Network model	Parameters	Accuracy
ResNet50	25.56M	84.6%
ResNet50-MPRB	21.10M	89.5%
MPC-STANet	27.25M	96.2%

**TABLE 5:** The influence of attention mechanisms on network accuracy.

Network model	Accuracy
ResNet50	84.6%
ResNet50-SE	85.9%
ResNet50-CMBA	87.8%
ResNet50- SCAM	90.1%
MPC-STANet	96.2%

**TABLE 6:** Performance evaluations of each disease stages.

Network model	Recall	F1-score	Precision
Non-Demented	97%	96%	97%
Very Mild Demented	95%	94%	95%
Mild Demented	97%	97%	98%
Moderate Demented	95%	93%	94%

**TABLE 7:** Evaluation indexes of the networks.

Network model	Recall	F1-score	Precision	mAP
ResNet50, He et al., 2016	83%	82%	85%	81%
VGG16, Toğaçar et al., 2021	80%	76%	77%	75%
U-Net, Hazarika et al., 2022	79%	75%	77%	73%
LeNet-5, Li et al., 2015	83%	82%	80%	75%
ADVIAN, Wang et al., 2019	84%	82%	85%	81%
MobileNet-SVM, Fei et al., 2022	90%	89%	89%	84%
DFNN, Huang et al., 2020	85%	82%	84%	81%
ResNet-STN, Sun et al., 2021	88%	89%	86%	83%
TReC, Xiao et al., 2021	91%	90%	92%	88%
Inception-v4, Bae et al., 2020	87%	90%	88%	85%
EfficientNetB0, Savaş, 2022	92%	94%	94%	92%
AlexNet, Hanmugam et al., 2022	77%	73%	75%	70%
GoogleNet, Hanmugam et al., 2022	84%	87%	86%	81%
MPC-STANet	96%	95%	96%	93%

**FIGURE 3:**
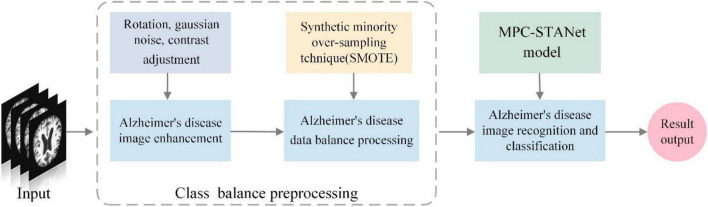
Principles of Alzheimer’s disease recognition.

Therefore, we propose a method in this study for recognizing disease stages of Alzheimer’s disease that combine class-balancing preprocessing and the MPC-STANet. The recognition principle is depicted in Figure 3. First, the minority classes are enhanced by flipping, adding noise and contrast adjustment, and then the class-balancing operation is achieved by SMOTE. Finally, the processed MRI dataset is input into the MPC-STANet for training and testing. To better extract the pathological features of Alzheimer’s disease, Dilated Convolution, Multi-Phantom Residual Block (including Multi-Conv Block and Multi-Identity Block), and Space Conversion Attention Mechanism are incorporated in the MPC-STANet to achieve better recognition accuracy.

## Materials and Methods

### Data Acquisition

Dataset is an important part of the field of pattern recognition and data mining. Since the main motivation of this study is to design a deep learning framework for Alzheimer’s disease classification, the adopted Alzheimer’s MRI dataset was created by researcher Sarvesh Dubey (Kaggle) and was collected from multiple websites, hospitals, and public repositories. The dataset consists of 896 MRI Mild Dementia images, 64 MRI Moderate Dementia images, 3,200 MRI Non-Dementia images, and 2,240 MRI Very Mild Dementia images, and the distribution of the number of MRI images in different stages of Alzheimer’s diseases is displayed in Table 8. All MRI images were preprocessed and resized to 128 × 128 pixels and saved in JPG format, and some of the images are depicted in Figure 1.

**TABLE 8:** Number distribution of Alzheimer’s disease dataset.

Disease type	Original number	Percentage
Non-Demented (ND)	3200	50%
Very Mild Demented (VMD)	2240	35%
Mild Demented (MD)	896	14%
Moderate Demented (MOD)	64	1%

### Class-Balancing Preprocessing Based on Data Expansion and SMOTE

As displayed in Table 8, the Alzheimer’s MRI dataset acquired has the problem of unbalanced distribution in terms of data volume, which will lead to the imbalanced learning effect of the neural network model, and the problems of overfitting and under-fitting exist simultaneously. To address this problem, we can expand the dataset with the help of data expansion methods and Synthetic Minority Oversampling Technique (SMOTE) technique to balance the data volume of the first four disease stages to improve the accuracy of the neural network model.

#### Data Expansion

**FIGURE 4:**
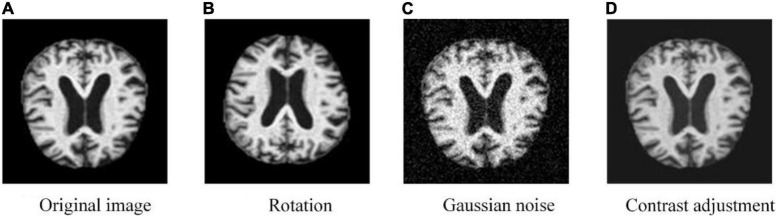
MRI image processed by augmentation methods.

Training the neural network model with more datasets allows it to learn more effective feature points to improve the recognition accuracy of the model, prevent overfitting, etc. We use MATLAB 2020b to flip the image, add random Gaussian noise, contrast adjustment, and other data expansion methods to expand the minority classes to improve the training effect of the neural network model. Also, the data expansion method is an important way to balance the data volume of different classes and the result images are shown in Figure 4.

#### Synthetic Minority Oversampling Technique

Table 8 displays the number of images in the dataset for the first four disease stages of Alzheimer’s disease, which indicates that the classes of the dataset are unbalanced. If a class-unbalanced dataset is used for prediction, the predictions tend to yield conclusions that are also biased, that is, the classification results will be biased toward the majority class. To address this problem, we apply synthetic minority oversampling technique (SMOTE) to this dataset, which addresses the classification imbalance in the dataset by randomly replicating the classes with fewer samples in the dataset to match the classes with more samples. We oversample the classes with fewer samples using the seeds of 42 random number generators, and Table 1 displays the distribution of the Alzheimer’s disease dataset after using the data expansion and SMOTE.

Suppose the number of the minority classes samples are *T*and set a sampling ratio to determine the magnification *N* according to the sample imbalance ratio so that the sample can be expanded by *N* times after sampling. The algorithm steps of the SMOTE are as follows:

Step1: Consider a sample *x* ∈ {1, …, *T*} in the minority class, calculate its distance to all samples in the minority classes based on the Euclidean distance, and select *K* the nearest neighbors.

(1) $$\left|x\right|=\sqrt{x_{1}^{2}+x_{2}^{2}+x_{3}^{2}+\ldots +x_{n}^{2}}$$

Step2: Randomly select a sample *B* from the *K* nearest neighbors and combine it with the original sample to synthesize a new sample according to the following formula.

(2) $$x_{new}=a+rand\left(0,1\right)\times \left|a-b\right|$$

Step3: Repeat Step 2 and Step 3 *N* times.

Step4: Repeat the above steps for *T* samples of the minority classes.

### ResNet50 Backbone

**FIGURE 5:**
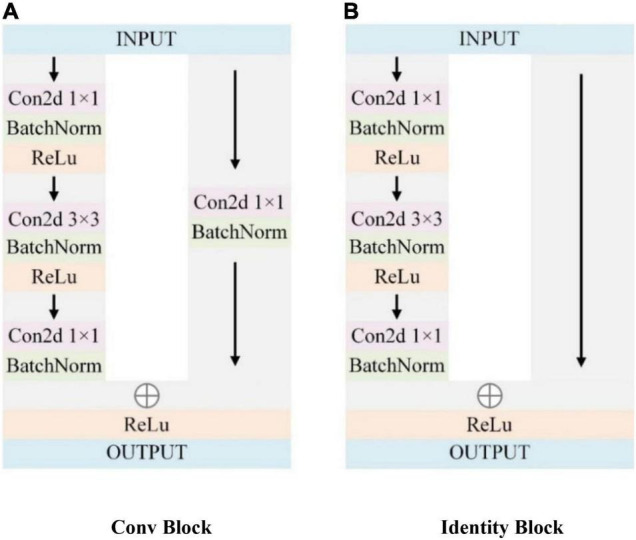
ResNet residual block.

ResNet50 constructs the deep network model as a shallow network model and an additional layer of self-mapping connects the trained shallow structure with the additional layer of self-mapping through residual units, transmits the input across layers through a shortcut, and then adds the output after convolution to achieve the effect of fully training the underlying network. ResNet50 has 6 STAGE (STAGE1∼ STAGE6), containing 49 convolutional layers and 1 fully connected layer. Among them, the 49 convolutional layers consist of two basic blocks. As shown in Figure 5, one is Identity Block, which has the same dimension of input and output, so it can be concatenated with more than one for deepening the network layers; the other basic block is Conv Block, which has an inconsistent dimension of input and output, so it cannot be concatenated consecutively and its role is to change the dimension of the feature vector.

The actual Alzheimer’s disease needs to be judged by looking at the pathological features such as the degree of enlargement of the sulcal gaps, and the degree of atrophy in the hippocampus region and medial temporal lobe. However, due to the small variation in MRI image features during the four disease stages: mild dementia (MID), moderate dementia (MOD), non-dementia (ND), and very mild dementia (VMD), more subtle pathological features need to be extracted to better discriminate. To extract more subtle pathological features, this study employs ResNet50, the winner of the ImageNet large-scale visual recognition competition in 2015, as the basic network. However, the properties of the Alzheimer’s MRI images are very different from those of ordinary images, and designing according to the pathological characteristics of Alzheimer’s disease can effectively improve the accuracy of the model. Therefore, we propose a recognition network of Alzheimer’s disease based on Multi-Phantom Convolution and Space Conversion Attention Mechanism (MPC-STANet), which is improved based on ResNet50.

### Recognition Network of Alzheimer’s Disease Based on Multi-Phantom Convolution and Space Conversion Attention Mechanism

**FIGURE 6:**
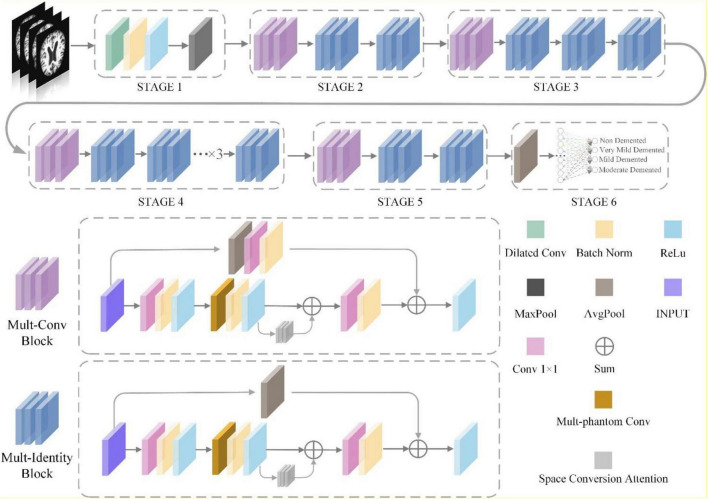
The t overall architecture of the MPC-STANe.

The MPC-STANet is upgraded based on ResNet50, and the network structure is depicted in Figure 6.

The feature extraction network of ResNet50 consists of 7 ×7 convolution and 3 ×3 maximum pooling layer (STAGE1), convolutional residual extraction network composed of Conv Block and Identity Block (STAGE2~STAGE5), average pooling layer, and fully connected layer (STAGE6). The MPC-STANet proposed in this study is based on ResNet50, using the Dilated Convolution (DC) instead of 7 ×7 convolution of STAGE1; changing the two basic blocks of Conv Block and Identity Block using Multi-Parallel Convolution (MPC) and averaging pooling layer, proposing Multi-Conv Block and Multi-Identity Block; adding the Space Conversion Attention Mechanism (SCAM) between the convolution blocks. This study improves the network structure, and more details will be provided in the following chapters.

#### Dilated Convolution

The pathological feature points of different disease stages of Alzheimer’s disease are obscure and scattered. To classify disease stages based on the Alzheimer’s MRI medical images more accurately, more effective subtle pathological features need to be extracted. ResNet50 uses a 7 × 7 convolution with a large perceptual field in STAGE1, which is sufficient for extracting features from common and ordinary images in the ImageNet database, but it is difficult to adequately consider the subtle pathological features of MRI. Therefore, to reduce the information loss during the extraction process and improve the recognition ability of the model, we employ Dilated Convolution (DC) to replace the 7 × 7 convolution in STAGE1. Dilated Convolution increases the perceptual field while maintaining the size of the feature map unchanged and does not cause problems such as information loss.

**FIGURE 7:**
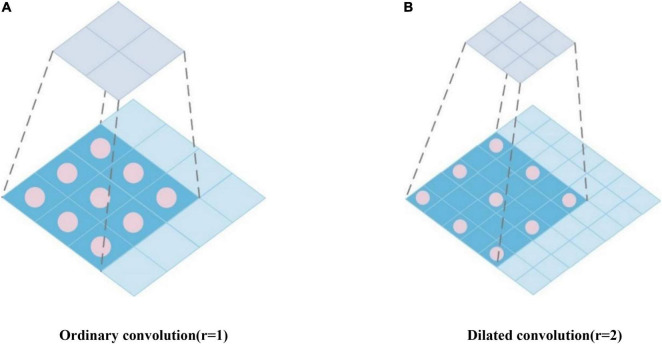
Perceptual field of ordinary convolution and extended convolution.

Dilated Convolution expands the perceptual field size of ordinary convolution by setting different dilation rates (*r*). Among them, *r* determines the interval size of the holes injected in the convolution. If *r* is too small, the range of the perceptual field is limited, and if *r* is too large, the features in the perceptual field lose some relevance. Dilated Convolution can be regarded as inserting a zero value of *r-1* into the convolution kernel during ordinary convolution. For ordinary convolution, the convolution kernel of 3 × 3 is calculated on the feature map, and the perceptual field of the new feature point is three, as depicted in Figure 7A. For the dilation convolution with dilation rate *r* = 2, one zero value is inserted between the 3 × 3 convolution kernels to obtain its perceptual field of five, as depicted in Figure 7B, which results in the equivalent of two ordinary 3 × 3 convolutions with only one computation.

Assuming that Dilated Convolution kernel is *k*×*k*, and the dilated rate is *r*, then the actual convolution kernel is:


(3) K=k+(k-1)×(r-1)

After Dilated Convolution process, the relationship between the size of the input and output feature maps is as follows:


(4) $$W_{2}=\frac{W_{1}+2p-r\times \left(k-1\right)-1}{s}+1$$

Among them, *W*_1_ and *W*_2_ represent the size of the input and output feature maps, respectively, *s* and *p* represent the step-size and the patch.

#### Multi-Conv Block and Multi-Identity Block Based on Multi-Phantom Convolution

ResNet50 residual block mainly consists of a linear branch (one 1 × 1 convolution layer and two 3 × 3 convolution layers) and a shortcut branch with 1 × 1 convolution, where the linear branch is used to extract feature information in the feature map and generate the output feature matrix; the shortcut branch uses 1 × 1 convolution to increase the number of channels and match the number of channels of the linear branch, which is used to avoid the problems of gradient disappearance and gradient explosion caused by network depth. Finally, the output feature matrixes of the two branches are summed to obtain the feature map of residual block output, and then the feature map is put into the Relu activation function to enhance the non-linearization of the model. However, due to the variety of structural changes in MRI images in different stages of Alzheimer’s disease such as the changes in the cerebral cortex, especially in the temporal and parietal regions, the features that appear tend to show only subtle differences; whereas the structure of the hippocampal region of suffering from Alzheimer’s disease is significantly changed in different stages.

**FIGURE 8:**
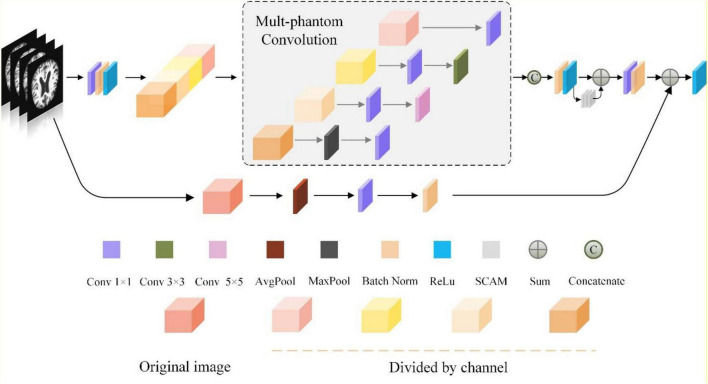
The structure of multi-phantom residual block.

To address this problem, we propose the Multi-Phantom Convolution (MPC) by borrowing the convolution by channel direction in the Inception network (Szegedy et al., 2016), and incorporating MPC into the residual block to propose Multi-Phantom Residual Block (MPRB), which has two blocks: Multi-Conv Block and Multi-Identity Block, to extract the features of more abundant characterization information in patients’ MRI. MPRB divides the feature matrix map output from 1 × 1 convolution into 4 parts of feature maps equally according to the channel direction, and then the feature maps of the different parts are extracted by different convolution and pooling operations for multi-scale feature extraction, and finally concatenated according to the channel direction. MPRB can extract more subtle pathological features; meanwhile, the MPRB reduces the training parameters and speeds up the convergence of the model when dividing the input feature maps and parallel convolution operations. In addition, it should be noted that the pathological features account for small regions of the whole MRI image and the proportion of information to be acquired is small. o avoid the redundancy of useless information, we add a 2 × 2 average pooling layer to integrate spatial information in the shortcut branch of MPRB, and the structure of MPRB is depicted in Figure 8. The average pooling layer has no parameters and does not change the global number of parameters while preventing overfitting at this layer.

The specific implementation process of Multi-Phantom Residual Block:

Step1: The input feature matrix is successively passed through the 1 × 1 convolution layer, batch norm layer, and Relu activation function, and takes the result as the input of step 2; the input feature matrix is successively passed through the average pool layer, 1 × 1 convolution layer and batch norm layer as the output of the shortcut branch.

Step2: The feature matrix of the linear branch is divided into four parts according to the channel direction.

Step3: The feature map I: passing through the 1 × 1 convolution layer; the feature map II: passing through the 1 × 1 convolution layer and 3 × 3 convolution layer successively; the feature map III: passing through the 1 × 1 convolution layer and 5 × 5 convolution layer successively; the feature map IV: passing through the maximum pool layer and 1 × 1 convolution layer successively.

Step4: The four feature maps are concatenated according to the channel direction and passed through the batch norm layer and Relu activate function.

Step5: The feature map output from step 4 with the feature map output through the Space Conversion Attention Mechanism (SCAM) are summed by pixels, and the sum result successively passes through the 1 × 1 convolution layer and batch norm layer.

Step6: Sum the linear branch (the output of step 5) with the shortcut branch, and pass through the Relu activate function.

#### Space Conversion Attention Mechanism

The visual attention mechanism is a brain signal processing mechanism that is unique to human vision. By quickly scanning the global image, human vision obtains the target region to be focused on, which is generally called the focus of attention, and then devotes more attention resources to this region to obtain more detailed information about the target to be focused on, while suppressing other useless information. The core goal of adding an attention mechanism to the network is essentially similar to the human selective visual attention mechanism, which also selects the more critical information for the current task goal from a multitude of information and ignores other redundant information to successfully improve the expressive power of the network.

The more popular attention mechanisms include SENet (Hu et al., 2018), CBAM (Woo et al., 2018), and Non-local Neural Networks (Wang et al., 2018), and many pieces of research combined with the recognition of attention mechanisms have achieved good recognition results, for example, Huang et al. (2020). proposed a brain tumor diagnosis system based on a differential feature neural network (DFNN), which mainly consists of an innovative differential feature map (DFM) block and a squeeze-and-excitation (SE) block. The experimental results indicated that the average accuracy of DFNN in classifying the brain as abnormal and normal on two databases was 99.2 and 98%, respectively. Xiao et al. (2021) proposed an early diagnosis method for pathological brain called the TReC, which imported the CBAM convolutional channel attention mechanism into the pre-trained ResNet residual block and replaced the fully connected layer with a new FC layer. The experimental results indicated an accuracy of 100% in the two-class classification task and an accuracy of 97.44% in the multi-class classification task. Sun et al. (2021) proposed a recognition method of the residual network (ResNet) combining space transformation network (STN) and non-local attention mechanism (non-local attention) to consider the long-range correlation in feature space, and successfully applied the method to the early diagnosis of Alzheimer’s disease, with the recognition accuracy of up to 97.1%, macroscopic accuracy of up to 95.5%, macroscopic recall of up to 95.3%, and macroscopic F1 value of up to 95.4%.

**FIGURE 9:**
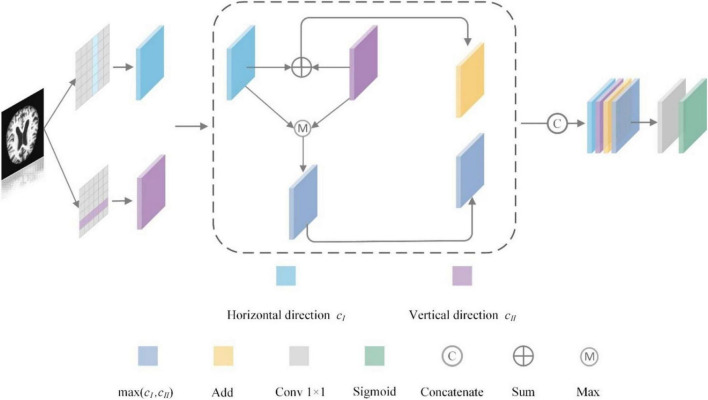
Space conversion attention mechanism structure.

The space dimension of the image refers to the height (H) and width (W) of the image, and C represents the feature channel of the image. The space attention mechanism pays attention to the importance of the space location features of the image, generating space attention coefficients for the output feature maps, and enhancing or suppressing different space location features according to the feature weights. The traditional space attention mechanism tends to focus on weight assignment in only one direction, which inevitably leads to the loss of important information in the image. For the Alzheimer’s MRI images, it is important to observe space changes in different disease stages, such as small changes in the cerebral cortex and structural changes in the hippocampal region, which are important for determining the stage of Alzheimer’s disease. Therefore, we propose a Space Conversion Attention Mechanism (SCAM) that assigns weights based on both vertical and horizontal directions, and the specific structure is depicted in Figure 9.

The Space Conversion Attention Mechanism is composed of three parts:

(a) The horizontal spatial attention mechanism is used to generate horizontally oriented weight coefficients for each row of features; the vertical spatial attention mechanism is used to generate vertically oriented weight coefficients for each column of features.

(5) $$c_{i}=\sum_{j=1}^{n}\frac{exp\left(e_{i,j}\right)}{\sum_{k=1}^{n}exp\left(e_{ik}\right)}h_{j}$$

Among them, *e*_*i,j*_ represents the weight coefficients assigned by the horizontal or vertical attention mechanism, pixel *j* represents the sequence feature, *i* represents the temporal features at a certain moment, and *h*_*j*_ represents the hidden layer information of the feature sequence *j*. *c*_I_ = {*c*_1_, *c*_2_ … *c*_*i*−1_, *c*_*i*_} represents the weight coefficients of the vertical attention mechanism in the feature sequence; *c*_Π_ = {*c*_1_, *c*_2_ … *c*_*i*−1_, *c*_*i*_} represents the weight coefficients of the horizontal attention mechanism in the feature sequence.

(b) To further expand the difference between the weight coefficients, we add the horizontal and vertical weight coefficients (addition strategy). for example, the small weight coefficient maybe 0.1 + 0.3 and the large weight coefficient maybe 0.8 + 0.9. In contrast, the difference between the summed weight coefficients is more obvious.

(6) $$add=c_{\text{I}}+c_{\Pi }$$

(c) To select the more interesting regions, we match the horizontal and vertical weight coefficients to find the maximum value (maximum strategy), which is used to complement the results of the second part of the weight coefficients [e.g., max(0.3, 0.8)].

(7) $$max=max\left(c_{\text{I}},c_{\Pi }\right)$$

Finally, we concatenate the weight coefficients calculated by the above strategy with *c*_*I*_ and *c*_Π_ through formula (6), and the concatenated results are passed through 1 × 1 convolution and sigmoid function to make the dimension of input and output consistent.


(8) $$SPA=concatenate\left(\left[c_{\text{I}},c_{\Pi },add,max\right]\right)$$

(9) $$\mathrm{Weight}=\sigma \left(F_{h}\left(\mathrm{SPA}\right)\right)$$

Among them, *SPA* represents the Space Conversion Attention Mechanism, *F*_*h*_ represents 1 × 1 convolution, σ represents the sigmoid function, and *Weight* represents the feature weights.

## Results and Analysis

### Experimental Environment and Settings

All of the trains and tests in this work are carried out on the same hardware and software platform. The hardware environment is Windows (64bit) operating system, Intel Core i7-9700U CPU, and 2080Ti GPU. The software programming environment for data expansion is MATLAB 2020b; The software programming environment for the MPC-STANet is Python 3.8.12, Pytorch 1.8.2, and CUDA 10.2. Considering the memory size of the GPU and the time of the experiment, we set the *Batchsize* to 32 for training and 8 for testing. The learning rate *Ir* was set to 10^−3^, and the *epochs* was set to 140. The Adam optimizer and Cross-Entropy Loss were used during training, and the incremental gradient descent was used as the training method. After class-balancing preprocessing, there were 12,600 MRI images in Alzheimer’s disease dataset. In this experiment, according to the ratio of 7:2:1, we divided the dataset into a training set, test set, and validation set for training and testing the MPC-STANet.

### Effectiveness Experiment of the Module

#### Effectiveness Experiment of Preprocessing

To verify whether the training with a class-balanced preprocessed dataset can improve the performance of the model and improve the recognition accuracy, we input the original dataset and the preprocessed dataset into ResNet50, ResNet50-SPAM, and the MPC-STANet, respectively, for experiments. Table 2 displays the recognition accuracy of the original dataset and the preprocessed dataset in the three networks. The results show that the recognition accuracy of the three networks in the preprocessed dataset is significantly higher than that of the original dataset. This is because the dataset is expanded by flipping, adding noise, and contrast adjustment, which increases the diversity of the dataset and avoids network coverage. The SMOTE algorithm is used to make the samples achieve class balance, to avoid the information learned during training to tend to the disease majority class. As a result, following preprocessing, the accuracy of the dataset has increased in all three models.

#### Effectiveness Experiment of Dilated Convolution

We used Dilated Convolution (DC) in STAGE 1 of the MPC-STANet. To verify the effect of the DC on classification performance, we conducted experiments on ResNet50, ResNet50-DC, and the MPC-STANet under the same test environment. Table 3 displays that using DC in ResNet50 can improve the recognition accuracy without changing the model parameters.

#### Effectiveness Experiment of Multiple-Phantom Residual Block Based on Multiple-Phantom Convolution

To verify the effect of the Multiple-Phantom Residual Block (MPRB) on model accuracy and parameter, we trained and tested ResNet50, ResNet50-MPRB, and the MPC-STANet using the same dataset. As displayed in Table 4, the experimental results show that ResNet50-MPRB with multiple-phantom residual blocks can greatly improve the accuracy of the network and reduce the number of parameters of the model.

#### Effectiveness Experiment of Space Conversion Attention Mechanism

To more intuitively understand the improvement of the network accuracy by Space Conversion Attention Mechanism (SCAM), we trained and tested ResNet50, ResNet50-SE, ResNet50-CMBA, ResNet50-SCAM, and the MPC-STANet, respectively, on the preprocessed dataset. Table 5 displays the accuracy of the networks with different attentions on the test set. The experimental results show that after using the attention mechanism, ResNet50-SE, ResNet50-CMBA, and ResNet50-SCAM improve 1.3, 3.2, and 5.5%, respectively, in terms of accuracy compared to ResNet50. The SCAM outperforms the other attention mechanisms in improving accuracy by considering the weight feature relationship in both horizontal and vertical directions. The accuracy of the MPC-STANet proposed in this study is 96.2%, which indicates that the Alzheimer’s MRI images features are deeply extracted, and the network is effective in recognizing.

#### Table Ablation Experiment

To fully validate the effectiveness of the method proposed in this study, we employed the same dataset and experimental environment, and only changed the parts that needed to be compared in each experiment. In this experiment, ResNet50 is selected as the backbone network, and one or more of the three methods, Dilated Conv (DC), Multi-Phantom Residual Block (MPRB), and Space Conversion Attention Mechanism (SCAM), are added to compare the effects of different schemes on model parameters and recognizing accuracy. The comparing results are displayed in Table 9.

**TABLE 9:** Comparison of recognition accuracy and parameters of different networks.

Network model	Parameters	Accuracy
ResNet50	25.56M	84.6%
ResNet50-DC	25.56M	86.7%
ResNet50-MPRB	21.10M	89.5%
ResNet50-SCAM	31.17M	90.1%
ResNet50-DC-MPRB	21.10M	93.3%
ResNet50-DC-SCAM	31.17M	93.8%
ResNet50-MPRB-SCAM	27.25M	94.6%
MPC-STANet	27.25M	96.2%

Based on the accuracy of the network, the accuracy of the MPC-STANet was higher than other networks, reaching 96.2%. When SCAM was applied to ResNet50, its accuracy improved by 5.5% compared to the original ResNet50. Similarly, ResNet50 using Dilated Conv or MPRB methods improved by 2.1 and 4.9%, respectively, compared to the original ResNet50. The preceding evidence indicates that all three methods are effective for increasing accuracy. And the solution of DC paired with MPRB or SCAM has the largest improvement in accuracy with 8.7 and 9.2%, respectively.

Based on the number of parameters of the network, the network with Dilated Conv is the same in terms of the number of parameters as the network that keeps a single variable, which is consistent with the principle that Dilated Convolution does not change the number of parameters. In terms of the number of parameters, ResNet50-MPRB is 4.46M less than ResNet50, demonstrating that the MPRB method aids in network compression.

#### Overall Evaluation of the MPC-STANet

In the same environment, the MPC-STANet has a more stable learning process and higher recognition accuracy than its backbone network ResNet50. The performance of the MPC-STANet on the four disease stages are depicted in Figure 2. In the confusion matrix, the numbers 0, 1, 2, and 3 represent the four disease stages, Non-Demented, Very Mild Demented, Mild Demented, and Moderate Demented, respectively. We tested the MPC-STANet using a total of 2,560 MRI test sets and displayed the test results in the confusion matrix. The diagonal of the confusion matrix is the number of correctly predicted images with a total of 2,462 MRI images. The overall recognition rate of the MPC-STANet is 96.2%. Table 6 displays the recognition rate of the four disease stages in the MPC-STANet. It can be seen that the highest precision of Mild Demented reached 98%, and that of Moderate Demented was only 94%.

#### Comparison With Other Networks

We employ four indexes, Recall, F1-score, Precision, and mAP to evaluate the performance of the MPC-STANet. The results are displayed in Table 7, The performance indexes of the MPC-STANet all surpass 90%, higher than those of other networks, indicating that this network is more advantageous in recognizing Alzheimer’s disease than other networks, and is better for classifying the disease stage.

## Discussion

In this study, we construct the MPC-STANet capable of discriminating the first four disease stages of Alzheimer’s disease and use the Alzheimer’s MRI images created by researcher Sarvesh Dubey as the dataset. We employ data expansion and SMOTE to perform class-balancing preprocessing of the dataset, and then input the preprocessed dataset into the MPC-STANet for recognition, and its average recognition accuracy reaches 96.25%. The experiments show that the combination of class-balancing preprocessing and MPC-STANet for the recognition of the first four disease stages of Alzheimer’s disease is effective and does not require operations such as numerous feature enhancement preprocessing or manual feature extraction, but the following explorations are needed: (1) The researcher who provided the Alzheimer’s MRI dataset did not provide any statistical information about patients and did not account for this condition, which raises doubts about our recommended approach. Therefore, Alzheimer’s disease datasets with detailed statistics need to be further considered in future explorations to be more convincing. (2) In the Space Conversion Attention Mechanism, we employ the maximum strategy to match the vertical and horizontal weight coefficients to select the regions of interest. However, we tend to ignore the data in the small value regions using the maximum value strategy, resulting in data loss. Therefore, it is worth thinking about considering both maximum and minimum values. (3) The actual data volume of Alzheimer’s disease collected in this study is not enough. In the future, the Alzheimer’s MRI image data should be further enriched to improve the generalization ability of the model.

## Conclusion

To address the problems of classification imbalance of the Alzheimer’s MRI datasets, small structural changes during different disease stages, small proportion of feature regions to the whole MRI image, and scattered features, we propose a novel method for recognizing different disease stages of Alzheimer’s disease based on class-balancing preprocessing and Multi-Phantom Convolution and Space Conversion Attention Mechanism recognition network (MPC-STANet). First, we perform class-balancing preprocessing on the Alzheimer’s MRI datasets using data expansion methods such as flipping, adding noise and contrast adjustment, and SMOTE. Then, we propose the MPC-STANet with ResNet50 as the backbone network. In the MPC-STANet, Dilated Convolution is used to increase the perceptual field of the network to recognize scattered feature regions, and Space Conversion Attention Mechanism is used to enhance feature extraction of subtle changes in the MRI Alzheimer’s image. Based on Multi-Phantom Convolution, Multi-Phantom Residual Block (including Multi-Conv Block and Multi-Identity Block) is proposed to extract subtle brain feature points. For the recognition of different disease stages of Alzheimer’s disease, the proposed MPC-STANet has higher recognition accuracy and a smaller number of parameters compared with the ResNet50 backbone network. The experimental results indicate that the recognition accuracy of the MPC-STANet is 96.2% and the number of parameters is only 1.69M higher than that of ResNet50.

Based on the detection of the disease stages of Alzheimer’s disease has been a hot research topic in the field of computer vision-aided diagnosis, The MPC-STANet can be used for disease stage recognition after acquiring the Alzheimer’s MRI dataset, which is significant for doctors to distinguish the disease and take corresponding treatment. Future research in this study will focus on how the network can handle complex structural brain features, how to enhance the extraction ability for subtle and scattered features, and how to handle datasets that are not preprocessed. In addition, we need to consider how to further optimize the structure of the network model to facilitate a more effective recognition of Alzheimer’s disease and delay the deterioration of this disease promptly.

## Data Availability Statement

The datasets presented in this study can be found in online repositories. The names of the repository/repositories and accession number(s) can be found in the article/supplementary material.

## Ethics Statement

Ethical review and approval was not required for the study on human participants in accordance with the local legislation and institutional requirements. Written informed consent for participation was not required for this study in accordance with the national legislation and the institutional requirements.

## Author Contributions

YL: methodology, writing-original draft preparation, conceptualization, and data curation. KT: software, data acquisition, and investigation. GZ: validation and project administration. AC: supervision and funding acquisition. WC: model guidance. RL: formal analysis and resources. LL: visualization and writing—review and editing. All authors contributed to the article and approved the submitted version.

## Conflict of Interest

The authors declare that the research was conducted in the absence of any commercial or financial relationships that could be construed as a potential conflict of interest.

## Publisher’s Note

All claims expressed in this article are solely those of the authors and do not necessarily represent those of their affiliated organizations, or those of the publisher, the editors and the reviewers. Any product that may be evaluated in this article, or claim that may be made by its manufacturer, is not guaranteed or endorsed by the publisher.

## References

[B1] Andrieu S. Coley N. Lovestone S. Aisen P. S. Vellas B. (2015). Prevention of sporadic Alzheimer’s disease: lessons learned from clinical trials and future directions. *Lancet Neurol.* 14 926–944. 10.1016/S1474-4422(15)00153-2 26213339

[B2] Bae J. B. Lee S. Jung W. Park S. Kim W. Oh H. (2020). Identification of Alzheimer’s disease using a convolutional neural network model based on T1-weighted magnetic resonance imaging. *Sci. Rep.* 10:22252. 10.1038/s41598-020-79243-9 33335244 PMC7746752

[B3] Beitz J. M. (2014). Parkinson’s disease: a review. *Front. Biosci.* 6 65–74. 10.4103/0028-3886.226451 24389262

[B4] Chawla N. V. Bowyer K. W. Hall L. O. Kegelmeyer W. P. (2002). SMOTE: synthetic minority over-sampling technique. *J. Artif. Intell. Res.* 16 321–357. 10.1186/1756-0381-6-16 24088532 PMC4016036

[B5] Chen J. E. Glover G. H. (2015). Functional magnetic resonance imaging methods. *Neuropsychol. Rev.* 25 289–313. 26248581 10.1007/s11065-015-9294-9 PMC4565730

[B6] De la Torre J. C. (1999). Critical threshold cerebral hypoperfusion causes Alzheimer’s disease? *Acta Neuropathol.* 98 424–436. 10.1007/s004010051044 10412794

[B7] Dubois B. Padovani A. Scheltens P. Rossi A. Dell’Agnello G. (2016). Timely diagnosis for Alzheimer’s disease: a literature review on benefits and challenges. *J. Alzheimers Dis.* 49 617–631. 10.3233/JAD-150692 26484931 PMC4927869

[B8] Fei Z. Yang E. Yu L. Li X. Zhou H. Zhou W. (2022). A Novel deep neural network-based emotion analysis system for automatic detection of mild cognitive impairment in the elderly. *Neurocomputing* 468 306–316.

[B9] Frisoni G. B. Fox N. C. Jack C. R. Jr. Scheltens P. Thompson P. M. (2010). The clinical use of structural MRI in Alzheimer disease. *Nat. Rev. Neurol.* 6 67–77. 20139996 10.1038/nrneurol.2009.215 PMC2938772

[B10] Guo L. Li N. Jia F. Lei Y. Lin J. (2017). A recurrent neural network based health indicator for remaining useful life prediction of bearings. *Neurocomputing* 240 98–109.

[B11] Hanmugam J. V. Duraisamy B. Simon B. C. Bhaskaran P. (2022). Alzheimer’s disease classification using pre-trained deep networks. *Biomed. Signal Process. Control* 71:103217.

[B12] Hazarika R. A. Maji A. K. Syiem R. Sur S. N. Kandar D. (2022). Hippocampus Segmentation Using U-Net Convolutional Network from Brain Magnetic Resonance Imaging (MRI). *J. Digit. Imaging.* [Epub ahead of print]. 10.1007/s10278-022-00613-y 35304675 PMC9485390

[B13] He K. Zhang X. Ren S. Sun J. (2016). “Deep residual learning for image recognition,” in *Proceedings of the IEEE Conference on Computer Vision and Pattern Recognition*, (Las Vegas, NV: IEEE), 770–778.

[B14] Hu H. Wei Y. Zhou Y. (2021). Product-harm crisis intelligent warning system design based on fine-grained sentiment analysis of automobile complaints. *Complex Intell. Syst.* 28 1–8. 10.1007/s40747-021-00306-z

[B15] Hu J. Shen L. Sun G. (2018). “Squeeze-and-excitation networks,” in *Proceedings of the IEEE Conference on Computer Vision and Pattern Recognition*, (Salt Lake City, UT: IEEE), 7132–7141.

[B16] Huang Z. Xu H. Su S. Wang T. Luo Y. Zhao X. (2020). A computer-aided diagnosis system for brain magnetic resonance imaging images using a novel differential feature neural network. *Comput. Biol. Med.* 121:103818. 10.1016/j.compbiomed.2020.103818 32568685

[B17] Ieracitano C. Mammone N. Bramanti A. Hussain A. Morabito F. C. (2019). A convolutional neural network approach for classification of dementia stages based on 2D-spectral representation of EEG recordings. *Neurocomputing* 323 96–107.

[B18] Kaggle (2022). *Alzheimer Detection and Classification.* San Francisco: Kaggle.

[B19] Lebedev A. V. Westman E. Van Westen G. J. Kramberger M. G. Lundervold A. Aarsland D. (2014). Random Forest ensembles for detection and prediction of Alzheimer’s disease with a good between-cohort robustness. *NeuroImage* 6 115–125. 10.1016/j.nicl.2014.08.023 25379423 PMC4215532

[B20] Li J. Luong M. T. Jurafsky D. Hovy E. (2015). When are tree structures necessary for deep learning of representations? *arXiv* [Preprint]. 10.48550/arXiv.1503.00185

[B21] Li Y. Wang Y. Wu G. Shi F. Zhou L. Lin W. (2012). Discriminant analysis of longitudinal cortical thickness changes in Alzheimer’s disease using dynamic and network features. *Neurobiol. Aging* 33 e15–e427. 10.1016/j.neurobiolaging.2010.11.008 21272960 PMC3086988

[B22] Lockhart S. N. DeCarli C. (2014). Structural imaging measures of brain aging. *Neuropsychol. Rev.* 24 271–289. 10.1007/s11065-014-9268-3 25146995 PMC4163469

[B23] López V. Fernández A. García S. Plade V. Herrera F. (2013). An insight into classification with imbalanced data: empirical results and current trends on using data intrinsic characteristics. *Inf. Sci.* 250 113–141.

[B24] Lu L. Zhang J. Xie Y. Gao F. Xu S. Wu X. (2020). Wearable health devices in health care: narrative systematic review. *JMIR mHealth uHealth* 8:e18907. 10.2196/18907 33164904 PMC7683248

[B25] Magnin B. Mesrob L. Kinkingnéhun S. élégrini-Issac M. Colliot O. Sarazin M. (2009). Support vector machine-based classification of Alzheimer’s disease from whole-brain anatomical MRI. *Neuroradiology* 51 73–83. 10.1007/s00234-008-0463-x 18846369

[B26] Maxwell A. E. Warner T. A. Fang F. (2018). Implementation of machine-learning classification in remote sensing: an applied review. *Int. J. Remote Sens.* 39 2784–2817.

[B27] Negin F. Rodriguez P. Koperski M. Kerboua A. Gonzàlez J. Bourgeois J. (2018). PRAXIS: towards automatic cognitive assessment using gesture recognition. *Expert Syst. Appl.* 106 21–35.

[B28] Nelson P. T. Alafuzoff I. Bigio E. H. Bouras C. Braak H. Cairns N. J. (2012). Correlation of Alzheimer disease neuropathologic changes with cognitive status: a review of the literature. *J. Neuropathol. Exp. Neurol.* 71 362–381. 10.1097/NEN.0b013e31825018f7 22487856 PMC3560290

[B29] Royce C. S. Hayes M. M. Schwartzstein R. M. (2019). Teaching critical thinking: a case for instruction in cognitive biases to reduce diagnostic errors and improve patient safety. *Acad. Med.* 94 187–194. 10.1097/ACM.0000000000002518 30398993

[B30] Sabuncu M. R. Konukoglu E. (2015). Clinical prediction from structural brain MRI scans: a large-scale empirical study. *Neuroinformatics* 13 31–46. 10.1007/s12021-014-9238-1 25048627 PMC4303550

[B31] Sarraf S. Tofighi G. (2016). Classification of alzheimer’s disease using fmri data and deep learning convolutional neural networks. *arXiv* [Preprint]. 10.48550/arXiv.1603.08631

[B32] Savaş S. (2022). Detecting the Stages of Alzheimer’s Disease with Pre-trained Deep Learning Architectures. *Arab. J. Sci. Eng.* 47 2201–2218.

[B33] Sun H. Wang A. Wang W. Liu C. (2021). An improved deep residual network prediction model for the early diagnosis of alzheimer’s disease. *Sensors* 21:4182. 10.3390/s21124182 34207145 PMC8235495

[B34] Szegedy C. Vanhoucke V. Ioffe S. Shlens J. Wojna J. (2016). “Rethinking the inception architecture for computer vision,” in *Proceedings of the IEEE Conference on Computer Vision and Pattern Recognition*, (Las Vegas, NV: IEEE), 2818–2826.

[B35] Toğaçar M. Cömert Z. Ergen B. (2021). Enhancing of dataset using DeepDream, fuzzy color image enhancement and hypercolumn techniques to detection of the Alzheimer’s disease stages by deep learning model. *Neural Comput. Appl.* 33 9877–9889.

[B36] Wang S. Tang C. Sun J. Zhang Y. (2019). Cerebral micro-bleeding detection based on densely connected neural network. *Front. Neurosci.* 13:422. 10.3389/fnins.2019.00422 31156359 PMC6533830

[B37] Wang X. Girshick R. Gupta A. He H. (2018). “Non-local neural networks,” in . *Proceedings of the IEEE Conference on Computer Vision and Pattern Recognition*, (Salt Lake City, UT: IEEE), 7794–7803. 10.3390/s21206873

[B38] Woo S. Park J. Lee J. Y. Kweon I. S. (2018). “Cbam: Convolutional block attention module,” in *Proceedings of the European Conference on Computer Vision (ECCV)*, (Cham: Springer), 3–19. 10.1371/journal.pone.0264551

[B39] Wu Y. Deng L. Li G. Zhu J. Shi L. (2018). Spatio-temporal backpropagation for training high-performance spiking neural networks. *Front. Neurosci.* 12:331. 10.3389/fnins.2018.00331 29875621 PMC5974215

[B40] Wu Y. H. Swaab D. F. (2005). The human pineal gland and melatonin in aging and Alzheimer’s disease. *J. Pineal Res.* 38 145–152. 10.1111/j.1600-079X.2004.00196.x 15725334

[B41] Xiao Y. Yin H. Wang S. H. Zhang Y. D. (2021). TReC: transferred ResNet and CBAM for Detecting Brain Diseases. *Front. Neuroinform.* 15:781551. 10.3389/fninf.2021.781551 35002667 PMC8733727

[B42] Young J. Modat M. Cardoso M. J. Mendelson A. Cash D. Ourselin S. (2013). Accurate multimodal probabilistic prediction of conversion to Alzheimer’s disease in patients with mild cognitive impairment. *NeuroImage* 2 735–745. 10.1016/j.nicl.2013.05.004 24179825 PMC3777690

[B43] Yu F. Koltun V. (2015). Multi-scale context aggregation by dilated convolutions. *arXiv* [Preprint]. 10.48550/arXiv.1511.07122

[B44] Zhao Y. Zhao B. (2013). Oxidative stress and the pathogenesis of Alzheimer’s disease. *Oxidative Med. Cell. Longev.* 2013: 316523. 10.1155/2013/316523 PMC3745981 23983897

